# The Common Task

**Published:** 1907-09-14

**Authors:** 


### THE COMMON TASK.

### The Best Food for Workers.

The recent Food Campaign, as it has oddly been called, has done something to open the eyes of the public to the futility of the meat-eating craze which has of recent years grown in intensity with the introduction of cheap meat from over the seas. The theory which to many housekeepers is an axiom not to be disputed, that large quantities of meat must be administered three times a day to people whose occupations make a great strain on their bodily and mental powers, melts away entirely in the light of a little investigation. To recognise that such workers require abundance of proteid, is quite another thing from admitting that the proteid should be obtained principally from meat. A list of all the articles containing valuable nitrogenous properties should be in the hands of every housekeeper when she is preparing the daily menu, and would not only help her to vary the diet of her household, but also to reduce her weekly bills. In some respects meat is unsuited to be the principal article of diet for busy people. It takes a great deal of digesting, it takes particular skill in cooking, and it is difficult to get it in perfect condition, that is, well-hung, tender, and thoroughly wholesome. The Bread and Food Reform League is doing good service in bringing the value of other forms of nourishment to the front, and we note with thankfulness that it is free from the reckless faddism which so often disfigures the utterances of otherwise sensible people on this topic.

### Nurses' Fees in America.

A good deal of discussion has been aroused on the subject of the large fees received by certain nurses in New York. As a rule patients are able to protect themselves from extortion by aid of a natural and healthy competition, and in England the custom of supplying nurses through co-operations tends to equalise the rate of payment. The difference between fees in London and New York is partly one of organisation, the two or three guineas a week received by the Englishwoman being pure profit, while the five guineas of the American has to provide her with the expense of living out. Even taking this into account the amount which the New York patient often has to pay seems very high, reaching occasionally a total of 35 dollars, or £7 a week. Obstetric nurses who have a good connection can, we believe, command fees approaching this figure even over here, but it would be for the ultimate disadvantage of nurses if fees ever came to be inflated in this country to such an extent that the nurse was regarded as an expensive luxury, to be dispensed with whenever possible, instead of the necessary assistant in even a slight illness. It is more important that the profession should be large and well esteemed, than that a small group of women should be enabled to secure fancy prices for their services.

